# Short time effects of two radiofrequency ablation methods on hypertrophic obstructive cardiomyopathy

**DOI:** 10.1002/clc.24217

**Published:** 2024-03-04

**Authors:** Yin‐ge He, Yong Dong, Shao‐hua Yang, Fan Yang, Jin‐Lei Yin, Hui‐qin Zhao, Yu‐jie Zhao

**Affiliations:** ^1^ Department of Cardiology Zhengzhou China

**Keywords:** endocardial radiofrequency septal ablation, hypertrophic obstructive cardiomyopathy, percutaneous intramyocardial septal radiofrequency ablation, systolic anterior motion

## Abstract

**Background:**

Radiofrequency ablation has been applied for the treatment of hypertrophic obstructive cardiomyopathy (HOCM). The two known procedures are percutaneous intramyocardial septal radiofrequency ablation (PIMSRA) and endocardial radiofrequency septal ablation (ERSA).

**Methods:**

This study presents a retrospective analysis of the PIMSRA and ERSA procedures in patients with drug‐refractory HOCM. A total of 28 patients participated in the study, with 12 receiving PIMSRA and 16 receiving ERSA. The objective of our study was to compare the short‐term effects of these two radiofrequency ablation procedures.

**Results:**

At the 30‐day follow‐up, the PIMSRA group demonstrated a greater reduction in left ventricular outflow tract peak gradient at rest compared to the ERSA group (22.25 [16.72] mmHg versus 47.75 [21.94] mmHg) (*p* < .01). The values for the PIMSRA group decreased from 99.33 (32.00) mmHg to 22.25 (16.72) mmHg (*p* < .01), while the ERSA group decreased from 97.75 (30.24) mmHg to 47.75 (21.94) mmHg (*p* < .01). Only the PIMSRA group exhibited a decrease in mitral regurgitation (MR). The area of MR decreased from 10.13 (4.12) mm^2^ to 3.65 (2.80) mm^2^ in the PIMSRA group (*p* < .01). Additionally, the PIMSRA group experienced reductions in left atrial diameter (LAD) and left ventricular ejection fraction (LVEF)%. The values for LAD changed from 43.58 (7.53) mm to 37.08 (6.92) mm (*p* = .03), and the values for LVEF% decreased from 65.75 (6.12) pg/mL to 60.83 (4.06) pg/mL (*p* = .03).

**Conclusion:**

In terms of the two types of radiofrequency ablation methods used in HOCM, it has been observed that PIMSRA demonstrates a more favorable early treatment effect compared to ERSA.

**Abbreviations**
ASA: alcohol septal ablation
C‐sept: coaptation‐septal
ERSA: endocardial radiofrequency septal ablation
ESV: end‐systolic volume
HCM: hypertrophic cardiomyopathy
HOCM: hypertrophic obstructive cardiomyopathy
ICE: intracardiac echocardiography
IVS: interventricular septum
LAD: left atrial diameter
LVEF: left ventricular ejection fraction
LVOT: left ventricular outflow tract
MR: mitral regurgitation
MV: mitral valve
NT‐proBNP: N‐terminal pro‐brain natriuretic peptide
NYHA: New York Heart Association
PIMSRA: percutaneous intramyocardial septal radiofrequency ablation
RV: right ventricle
SAM: systolic anterior motion
SE: stress echocardiography
SM: surgical myectomy
SRT: septal reduction therapy
TTE: transthoracic echocardiography

## 1 INTRODUCTION

Hypertrophic cardiomyopathy (HCM) is defined as having left ventricular hypertrophy without any additional potential cardiac, systemic, syndromic, or metabolic disease that could be considered causal.^1^ Hypertrophic obstructive cardiomyopathy (HOCM) is a subtype of HCM that has an obstruction in the left ventricular outflow tract (LVOT), which is present in around 70% of HCM patients.^2^

The main objective in the treatment of HOCM is to decrease the amount of hypertrophy in the basal septum and to alleviate the systolic anterior motion (SAM) of the mitral valve (MV) leaflet.^3^, ^4^ Patients with drug‐refractory HOCM and have a resting or provoked LVOT gradient of 50 mmHg or higher, as well as being in functional class III or IV according to the New York Heart Association (NYHA), are primarily treated with septal reduction therapy (SRT).^5^ The traditional methods for SRT include surgical myectomy (SM) or alcohol septal ablation (ASA). SM effectively reduces LVOT obstruction and carries a low risk of mortality. However, it may not be accessible in all medical centers.^6^, ^7^ ASA is an alternative option for patients who are not suitable candidates for SM, but the success of ASA heavily relies on the patient's specific coronary anatomy needed for the ablation procedure, and around 15% of patients do not have a suitable septal vessel. Additionally, nearly 17% of patients require a permanent pacemaker implantation following ASA.^8^, ^9^

The radiofrequency ablation method can also be used to implement SAT in HOCM patients, which includes two methods: endocardial radiofrequency septal ablation (ERSA) and percutaneous intramyocardial septal radiofrequency ablation (PIMSRA).^10^, ^11^, ^12^, ^13^ ERSA built a three‐dimensional model of LVOT and SAM region under the guidance of Carto, the ablation creates a localized myocardial scar at the SAM‐septum contact point, which can disrupts the feedback mechanism and soon reduces the LVOT gradient.^14^, ^15^, ^16^ Liu et al.^13^, ^17^ change the HOCM radiofrequency ablation road to ablate the inner layer of interventricular septum (IVS) myocardium through the apical approach, this modification aims to avoid damage to the conduction system and reduce the dependency on the coronary septal branches.^12^ The objective of this study is to compare the short‐term effectiveness of the two different radiofrequency ablation method on HOCM patients.

## 2 PATIENTS AND METHODS

### 2.1 Patients selection

Between July 2019 and June 2023, we conducted a research study on 195 patients with HOCM who qualified for SRT according to the 2014 ESC guidelines. Among these patients, 155 opted for ASA or SM, while 12 made the final decision not to undergo any surgical intervention. Out of the remaining 28 patients, 12 received PIMSRA treatment, and the remaining 16 underwent ERSA. The study protocol was approved by the ethics committee of Zhengzhou No. 7 People's Hospital.

### 2.2 Imaging study

LVOT was measured using transthoracic echocardiography (TTE) at rest in all patients, both before and 30 days after PIMSRA and ERSA procedures during follow‐up. The TTE indicators included the SAM phenomenon, mitral regurgitation (MR), left atrial anterior and posterior diameter (LAD), left ventricular ejection fraction (LVEF) percentages, The LVOT peak gradient at rest, thickness of IVS, and the thickest location on the IVS, which were all measured at baseline before the PIMSRA or ERSA procedures and again at 30‐day after the procedures. Based on the guidelines from the American Society of Echocardiography,^18^ LVEF was derived using the bi‐plane Simpson method.

### 2.3 PIMSRA and ERSA procedure

#### 2.3.1 PIMSRA procedure

**Figure 1:**
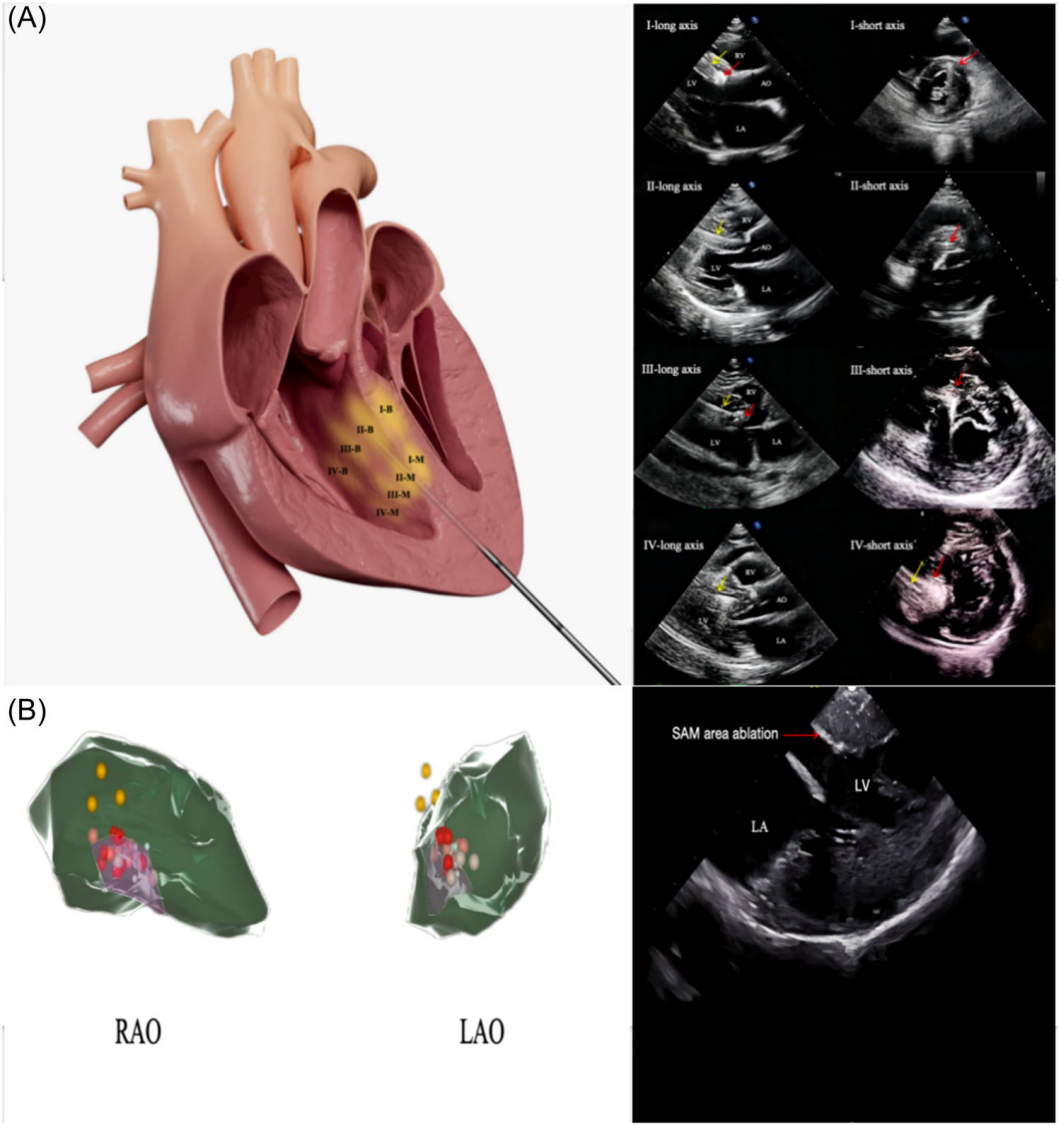
Surgical schematic and ultrasound images of PIMSRA and ERSA. (A) The ablation area of PIMSRA, which is shown in the left image, is divided into four regions within the IVS. To maximize the ablation area of the IVS, both the basal and middle parts of each region must be ablated. The basal part of regions I‐IV of the IVS septum is denoted as (I‐IV)‐B, while the middle part is represented as (I‐IV)‐M. The right image displays the long‐axis and short‐axis images of the left ventricle of regions I‐IV in TTE. The ablation area is indicated by the red arrow, and the ablation needle is indicated by the yellow arrow. (B) The LAO and RAO views of the CARTO display present the SAM area of the posterior IVS in pink, the left bundle branch in yellow, and the ablation area in red. The ICE screen shows a clear echo of the ablation site in the zone where SAM is visible. ERSA, endocardial radiofrequency septal ablation; IVS, interventricular septum; LAO, left anterior oblique; PIMSRA, percutaneous intramyocardial septal radiofrequency ablation; RAO, right anterior oblique; SAM, systolic anterior motion; TTE, transthoracic echocardiography.

Patients who underwent PIMSRA were subjected to electrocardiography, blood pressure monitoring, and oxygen saturation monitoring after general anesthesia. The transapical intramyocardial approach with a radiofrequency electrode needle was used to treat the hypertrophied septum (Figure 1A). Color Doppler TTE was used to avoid damaging blood vessels. The high‐frequency alternating current generated heat in the myocardial cells, with temperatures potentially exceeding 80°C.^12^ The needle remained cool thanks to a closed circuit water cooling system, and the radiofrequency machine would automatically turn off if tissue impedance increased by 150%. Lidocaine treatment was provided in cases of prolonged heart block or tachyarrhythmia, and ablation was paused until a normal rhythm returned. To protect the cardiac conduction system, ablation was performed on the basal and middle parts of both the anterior and posterior septum (Figure 1A). Pressure was applied locally for 5–10 minutes to stop bleeding, and the patient's vital signs and symptoms were carefully monitored using echocardiography to check for pericardial effusion. A final TTE hemodynamic assessment was conducted after observing the patient for at least 15 minutes.

#### 2.3.2 ERSA procedure

Conscious sedation using a combination of fentanyl and midazolam was administered throughout all procedures, with close monitoring of invasive arterial blood pressure. The SoundStar™ catheter was inserted through the left femoral vein and guided into the right ventricle (RV) using a phased‐array probe. This probe was used to determine the geometries of intracardiac echocardiography (ICE) in the RV, left ventricle, and aorta. ICE was also used to locate and monitor the ablation of the SAM (Figure 1B). Accurate identification of the endocardial borders and aortic cusps allowed for their transfer to the CARTO system for hypertrophic septum analysis. The His bundle was located using a quadripolar catheter (yellow dot in Figure 1B), along with back‐up RV pacing. Heparin was administered intravenously to maintain activated clotting time, while the location of the His bundle and left bundle was indicated on the CARTO shell.^19^ Radiofrequency energy was delivered using either a Navistar THERMOCOOL catheter or a Smartouch catheter, and the ablation points were marked (red or pink dots in Figure 1B, different colored dots represent different ablation index values), combined with CARTO and intracardiac echo navigation, targeting the area where the SAM and septal contact occurred (pink area in Figure 1B). Precautions were taken to avoid harming the His bundle, left bundle, and fascicles during the ablation process. The procedural endpoint was defined as either a reduction in the LVOT gradient by more than 50% or complete ablation of the area where the SAM and septal contact occurred. Methylprednisolone was administered for 3 days after the procedure to reduce edema.

### 2.4 Statistical analysis

Continuous variables were presented as mean ± standard deviation or median (interquartile range), while categorical variables were shown as frequencies and proportions. The two‐sample Student *t*‐test was used to analyze comparisons of clinical outcomes between patients who underwent ERSA and PIMSRA procedures for continuous variables, while the *χ* ^2^ test or Fisher exact test was used for categorical variables, as appropriate. The one‐way analysis of variance test was used to analyze comparisons between multiple groups. Pearson correlation coefficients were used to estimate correlations between continuous variables. Statistical significance was set at a two‐tailed *p*‐value of .05. All analyses were performed using Prism (version 9).

## 3 RESULTS

### 3.1 Baseline clinical characteristics

The baseline characteristics of 28 patients who underwent PIMSRA and ERSA procedures for HOCM are presented in Table 1. The ERSA group had a higher proportion of male patients compared to the PIMSRA group, a difference that was statistically significant (*p* = .02). Additionally, the ERSA group showed elevated levels of uric acid (*p* = .01) and creatinine (*p* = .04) compared to the PIMSRA group. Notably, patients with the thickened type of anterior ventricular septum (intermediate part of the anterior ventricular septum) were more likely to undergo SAM area ablation (ERSA), showing a significant association (*p* < .01). Conversely, in the PIMSRA group, the thickened type of basal ventricular septum was more prevalent (*p* < .01).

**Table 1:** Baseline characteristics between the PIMSRA group and ERSA group.

Baseline characteristics	PIMSRA	ERSA	*p*‐Value
Age (year)	55.33 (8.78)	51.19 (12.94)	.35
Male (%)	33.33	81.25	.02
BMI (kg/m^2^)	25.34 (2.96)	26.57 (3.54)	.34
Hypertension (%)	58.33	43.75	.70
Diabetes (%)	0.00	14.28	.49
CAD (%)	25.00	31.25	.99
AF (%)	16.66	25.00	.67
LAD (mm)	41.50 (5.55)	41.13 (6.60)	.88
LVEDd (mm)	44.00 (3.89)	44.31 (4.37)	.85
LVOT Gradient at rest (mmHg)	99.33 (32.00)	97.75 (30.24)	.89
LVOT Outflow velocity (cm/s)	493.30 (83.86)	487.40 (79.59)	.85
LVEF (%)	65.75 (6.12)	67.44 (6.93)	.50
ASA before (%)	90.09	23.07	.61
CK‐MB (ng/mL)	10.2 (12.64)	15.33 (8.27)	.20
NT‐proBNP (pg/mL)	1655 (1046)	2066 (1758)	.48
Creatinine	57.92 (11.03)	67.88 (13.53)	.04
Uric acid	285.6 (121.0)	409.8 (120.90	.01
Thickest location			
BAVS (%)	91.66	31.25	<.01
IAVS (%)	9.09	68.75	<.01
Thick of ventricular free wall (%)	100.00	100.00	–
Thickness of IVS	21.58 (2.93)	24.13 (4.41)	.09
SAM (%)	83.33	94.00	
Mitral regurgitation, cm^2^	10.13 (4.13)	5.60 (4.31)	<.01
Angina pectoris (%)	75.00	93.75	.28
Syncope/presyncope (%)	33.33	18.75	.42
Palpitation (%)	33.33	18.75	.42
NYHA functional class III‐IV (%)	25.00	18.75	.99
LVOT diameter (%)	19.92 (2.02)	19.94 (2.67)	.98
SBP (mmHg)	121.60 (10.85)	119.8 (11.91)	.68
DBP (mmHg)	77.92 (6.73)	74.75 (9.51)	.33

Abbreviations: AF, atrial fibrillation; ASA alcohol ablation; BAVS, basal part of anterior ventricular septum; BMI, body mass index; CAD, coronary heart disease; CK‐MB, creatine kinase MB; DBP, diastolic blood pressure; ERSA, endocardial radiofrequency septal ablation; IAVS, intermediate part of anterior ventricular septum; IPVS, intermediate part of posterior ventricular septum; IVS, interventricular septum; LAD, left atrial anterior and poster diameter; LVEDd, left ventricular end‐diastolic diameter; LVEF, left ventricular ejection fraction; LVOT, left ventricular outflow tract; NT‐proBNP, N‐terminal brain natriuretic peptide; NYHA, New York Heart Association; PIMSRA, percutaneous intramyocardial septal radiofrequency ablation; SAM, systolic anterior motion; SBP, systolic blood pressure.

### 3.2 Outcomes of clinical events

Table 2 presents the procedural, in‐hospital, and 30‐day clinical events for 28 patients. The occurrence rate of pericardial effusion is higher in the PIMSRA group compared with that in the ERSA group, pericardiocentesis drainage is required for two patients. One patient required a permanent pacemaker after the procedure. Another patient experienced persistent ventricular tachycardia during the operation, but sinus rhythm was restored after receiving lidocaine treatment. In the ERSA group, after the procedure, two patients developed left bundle branch block. One patient required a temporary pacemaker after the procedure and another patient required a permanent pacemaker. Additionally, one patient experienced persistent ventricular tachycardia during the operation.

**Table 2:** Presents the procedural, in‐hospital, and 30‐day clinical events.

	PIMSRA *n* = 12 (%)	ERSA *n* = 16 (%)	*p*‐Value
Pericardial effusion	6 (50.0)	0 (0)	.01
Requiring surgery	0 (0)	0 (0)	–
Requiring pericardiocentesis	2 (16.7)	0 (0)	.17
Stroke	0 (0)	0 (0)	–
RBBB	0 (0)	0 (0)	–
LBBB	0 (0)	2 (12.5)	.49
Temporary pacemaker in procedure	0 (0)	1 (6.3)	.99
Permanent pacemaker after the procedure	1 (8.3)	1 (6.3)	.99
Accelerated idioventricular rhythm	0 (0)	0 (0)	–
Ventricular tachycardia	1 (8.3)	1 (6.3)	.99

*Note*: For the PIMSRA group, half of the patients developed pericardial effusion after the procedure, while only two of them required pericardiocentesis. One patient developed ventricular tachycardia after the procedure and one patient developed degree III atrioventricular block and was fitted with a permanent pacemaker. For the ERSA group, a quarter of patients experienced complications, two patients developed LBBB and the other two patients had temporary and permanent pacemakers, respectively.

Abbreviations: ERSA, endocardial radiofrequency septal ablation; LBBB, left bundle branch block; PIMSRA, percutaneous intramyocardial septal radiofrequency ablation; RBBB, right bundle branch block.

### 3.3 Clinical outcome of PIMSRA and ERSA

#### 3.3.1 Outcome of PIMSRA procedure

After the PIMSRA procedure, the mean (SD) LVOT peak gradient at rest significantly decreased from 99.33 (32.00) mmHg to 22.25 (16.72) mmHg (*p* < .01) at the 30‐day follow‐up. Furthermore, the MR decreased from 10.13 (4.12) mm^2^ to 3.65 (2.80) mm^2^ (*p* < .01). The IVS thickness did not exhibit a significant decrease from 21.58 (2.93) mm to 19.83 (2.69) mm (*p* = .14). N‐terminal probrain natriuretic peptide (NT‐proBNP) levels decreased from 1719.00 (887.90) pg/mL to 797.5 (487.2) pg/mL (*p* < .01), while LVEF% decreased from 65.75 (6.12) pg/mL to 60.83 (4.06) pg/mL (*p* = .03). The number of SAM decreased from 10 to 4 (*p* = .03), and the LAD decreased from 43.58 (7.53) mm to 37.08 (6.92) mm (*p* = .03). Significant improvement was observed in the symptoms of angina pectoris (*p* < .01), although certain improvements were noted in other symptoms such as syncope, palpitation, and heart failure (NYHA III‐IV). However, these improvements did not achieve statistical significance, as indicated in Table 3. The systolic blood pressure (SBP) fluctuates between 121.6 (10.85) mmHg and 135.6 (20.42) mmHg before and after the procedure, and the observed change in SBP is considered statistically significant (*p* = .04). Similarly, there is a reduction in diastolic blood pressure (DBP) from 77.92 (6.73) mmHg to 71.42 (10.11) mmHg before and after the procedure. However, the difference in DBP is not determined to be statistically significant (*p* = .07).

**Table 3:** Radiofrequency ablation characteristics between the PIMSRA group and the ERSA group.

	PIMSRA			ERSA		
	Before procedure	30 days follow‐up	*p*‐Value	Before procedure	30 days follow‐up	*p*‐Value
Peak gradient of LVOT (mmHg)	99.33 (32.00)	22.25 (16.72)	<.01	97.75 (30.24)	47.75 (21.94)	<.01
MR (cm^2^)	10.13 (4.12)	3.65 (2.80)	<.01	5.60 (4.30)	4.68 (4.61)	<.01
NT‐proBNP (pg/mL)	1719.00 (887.90)	797.5 (487.2)	<.01	**–**	**–**	–
LVEF (%)	65.75 (6.12)	60.83 (4.06)	.03	67.44 (6.93)	64.63 (5.64)	.21
SAM (person)	10	4	.03	15	12	.33
LAD (mm)	43.58 (7.53)	37.08 (6.92)	.03	41.06 (6.65)	42.81 (6.47)	.45
IVS (mm)	21.58 (2.93)	19.83 (2.69)	.14	24.13 (4.41)	22.63 (4.81)	.36
Angina pectoris (person)	10	1	<.01	16	11	.04
Syncope	7	2	.09	3	1	.59
Palpitation	6	4	.68	6	5	.99
NYHA III‐IV	3	2	.99	3	2	.99

Abbreviations: ERSA, endocardial radiofrequency septal ablation; IVS, interventricular septum; LAD, left atrial anterior and poster diameter; LVEF, left ventricular ejection fraction; LVOT, left ventricular outflow tract; MR, mitral regurgitation; NT‐proBNP, N‐terminal probrain natriuretic peptide; NYHA, New York Heart Association; PIMSRA, percutaneous intramyocardial septal radiofrequency ablation; SAM, systolic anterior motion.

#### 3.3.2 Outcome of ERSA procedure

A significant decrease in the LVOT peak gradient at rest, from 97.75 (30.24) mmHg to 47.75 (21.94) mmHg (*p* < .01), was observed after the ERSA procedure at the 30 days follow‐up. However, there was no significant improvement in MR, as there was only a slight change from 5.60 (4.30) mm^2^ to 4.68 (4.61) mm^2^ (*p* = .56). No significant differences were found in the thickness of IVS (from 24.13 [4.41] mm to 22.63 [4.81] mm, *p* = .36), LAD (from 41.06 [6.65] mm to 42.81 [6.47] mm, *p* = .45), and LVEF% (from 67.44 [6.93] to 64.63 [5.64], *p* = .21). Additionally, there was no significant difference in the number of SAM (from 15 to 12, *p* = .33) according to Table 3.

The average SBP before the procedure for ERSA is 119.80 (11.91) mmHg, while after the procedure, the average SBP is 122.60 (11.85) mmHg. The statistical analysis shows that there is no significant change in SBP (*p* = .51). Before the procedure, the average DBP is 74.75 (9.51) mmHg, which decreases to 73.44 (12.62) mmHg after the procedure. However, the statistical analysis reveals that the change in DBP is not statistically significant (*p* = .74).

### 3.4 Comparison between PIMSRA and ERSA

#### 3.4.1 The LVOT peak gradient at rest between PIMSRA and ERSA

**Figure 2:**
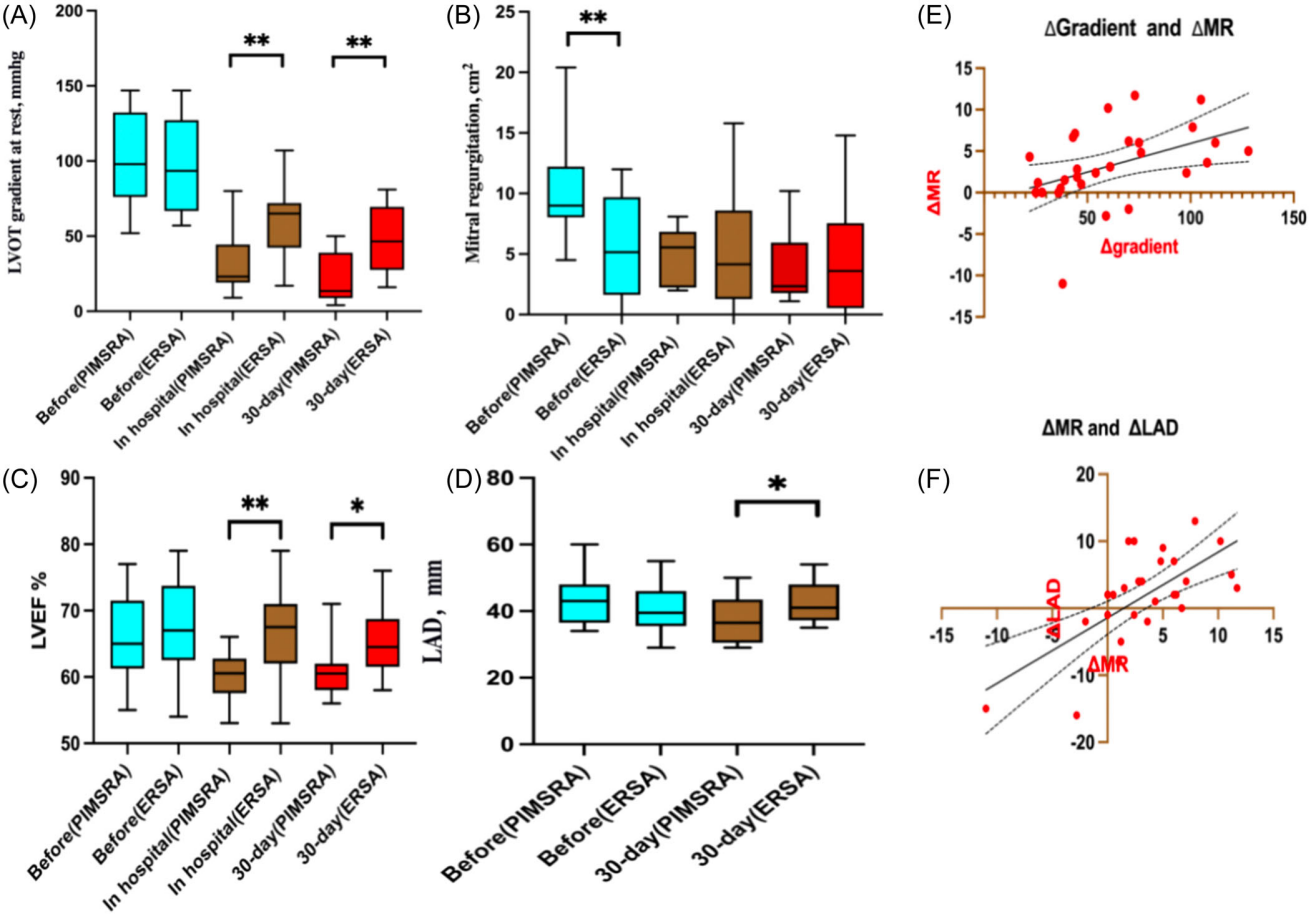
Comparison of PIMSRA and ERSA postoperative indices, along with the linear correlation between Δgradient and ΔMR, as well as the relationship between ΔMR and ΔLAD. (A) Comparison of the resting peak gradient of the LVOT before the procedure, in‐hospital, and 30 days after for PMISRA and ERSA. (B) Comparison of the mitral regurgitation before the procedure, in‐hospital, and 30 days after for PMISRA and ERSA. (C) Comparison of the LVEF before the procedure, in‐hospital, and 30 days after for PMISRA and ERSA. (D) Comparison of the LAD before the procedure, in‐hospital, and 30 days after for PMISRA and ERSA (**p < .01, *p < .05). (E) There is a linear correlation between the LVOT peak Δgradient and ΔMR. When the Δgradient gets higher, so does the ΔMR. (F) There is a linear correlation between the ΔMR and ΔLAD. When the ΔMR gets higher, so does the ΔLAD. ERSA, endocardial radiofrequency septal ablation; LAD, left atrial anterior and poster diameter; LVEF, left ventricular ejection fraction; LVOT, left ventricular outflow tract; MR, mitral regurgitation; PIMSRA, percutaneous intramyocardial septal radiofrequency ablation.

The LVOT peak gradient at rest was the same between the PIMSRA and ERSA groups before the two procedures [99.33 [32.00] mmHg vs. 97.75 [30.24] mmHg, respectively, *p* = .89]. However, the PIMSRA group exhibited a lower LVOT peak gradient at rest after the procedure compared to the ERSA group while still in the hospital (31.83 [21.37] mmHg vs. 59.69 [24.53] mmHg, respectively, *p* < .01), as well as during the 30 days follow‐up (22.25 [16.72) mmHg vs. 47.75 [21.94] mmHg, respectively, *p* < .01) (refer to Figure 2A).

#### 3.4.2 The MR between PIMSRA and ERSA

MR was different between PIMSRA and ERSA before the two procedures, with a mean of 10.13 (4.12) mm^2^ and 5.60 (4.30) mm^2^, respectively (*p* < .01). However, there was no significant difference in MR between PIMSRA and ERSA after the procedure, as measured both in hospital (4.70 [2.31] mm^2^ vs. 5.07 [4.45] mm^2^, *p* = .79) and during the 30 days follow‐up (3.65 [2.80] mm^2^ vs. 4.68 [4.61] mm^2^, *p* = .50) see Figure 2B.

#### 3.4.3 The LVEF% between PIMSRA and ERSA

The LVEF% before the PIMSRA and ERSA procedures were 65.75 (6.12) and 67.44 (6.93), respectively (*p* = .50). After the procedure and during hospitalization, the mean values of LVEF% were 60.00 (3.62) and 66.81 (7.09), respectively for the PIMSRA and ERSA procedures (*p* < .01). During the 30 days follow‐up period, a significant difference was observed between PIMSRA and ERSA, with mean values of 60.83 (4.06) and 65.25 (5.02), respectively (*p* = .02), as shown in Figure 2C.

#### 3.4.4 The LAD between PIMSRA and ERSA

Before the procedure, no significant was showed in LAD between the PIMSRA and ERSA group, the means were 43.58 (7.53) mm and 41.06 (6.65) mm, respectively (*p* = .35). During the 30‐day follow‐up period. PIMSRA group exhibited lower LAD, the means were 37.08 (6.92) mm and 42.81 (6.47) mm, respectively (*p* = .03). Figure 2D displays these results.

### 3.5 ΔGradient and ΔLAD are linearly correlated with ΔMR (Δ means the difference between the indicators before and in 30 days follow‐up)

Out of the 28 patients, it was observed that the gradients after two types of radiofrequency ablation were significantly different. Based on this finding, a linear relationship was discovered between Δgradient and ΔMR (*p* = .02) (as seen in Figure 2E), as well as between ΔMR and ΔLAD (as seen in Figure 2F) (*p* < .01).

## 4 DISCUSSION

Two different radiofrequency ablation methods for treating HOCM were discussed in this study. PIMSRA ablation, which enters through the apex of the heart, targets the inner myocardium of the ventricular septum. On the other hand, ERSA ablation targets the endocardium of the SAM region.

In cases of HOCM, ventricular septal hypertrophy irregularities are commonly observed, and the specific area of hypertrophy can vary greatly.^20^ The use of ASA and septal SM can potentially harm the conduction system, thus increasing the risk of ventricular septal perforation. Thanks to ultrasound guidance or labeling the conduction bundle potential, both PIMSRA and ERSA can better avoid damaging the conduction bundles. PIMSRA completely eradicates these possible complications by employing ultrasound guidance and guaranteeing that the ablation procedure is performed at a distance from the conduction tract. The procedure can bypass both the larger coronary artery and the conduction tract. This ensures a safer and more effective ablation process overall.

Among the two ablation methods, PIMSRA has proven to have a more effective ablation effect. This is evident through a low LVOT peak gradient at rest and a greater improvement of SAM‐mediated MR. We have witnessed PIMSRA in 12 cases and compared it to previous treatment reports. Our observations have indicated that the decrease in LVOT peak gradient at rest is comparable to the findings reported by Liu et al.^21^ Moreover, it is similar to the observed effect of SM and superior to that of ASA.^22^, ^23^

We observed not only a significant decrease in the LVOT peak gradient at rest but also an improvement in MR, accompanied by a decrease in LVEF%. However, the early stages of ablation cannot bring about a rapid thinning of the IVS. The initial change in the LVOT gradient is primarily caused by the weakening of the contractile force of the IVS, which can be attributed to coronary microcirculation and myocardial injury in the septum.^24^ Therefore, for patients with HOCM, the composition of the obstruction has two important factors: (1) the obstruction pathway, (2) the strong contraction force of the myocardium. Whether it is PIMSRA or ERSA, the relief of obstructed pathway is not accompanied in the early procedure, as the thinning of the IVS is not significant. Hence, we hold the view that the decline in myocardial contractility is the primary cause of the early reduction in LVOT gradient during radiofrequency ablation.

The muscle fibers in the mid‐wall layer are arranged in a circular orientation around the short axis. When this layer of muscle contracts, it causes both radial and circumferential shortening. The radial strain (RS) and circumferential strain (CS) of ablated segments in the IVS were significantly decreased immediately after PIMSRA,^24^ while no significant changes of longitudinal strain (LS) were observed. The impact of heart size on LVEF% should be emphasized, as a decrease in LVEF% indicates a decrease in end‐diastolic volume or an increase in end‐systolic volume (ESV). During systole, as RS and CS decrease, the diameter of the ventricular cavity increases, resulting in an increase in ESV and a subsequent decrease in LVEF%.^25^, ^26^, ^27^ Furthermore, there was a decrease in NT‐ProBNP, indicating improved cardiac function and reduced ventricular tension. This could provide evidence of improved diastolic function after PIMSRA, as well as a decrease in systolic function without affecting cardiac function. Despite a reduction in LVEF%, the outer myocardia remained unaffected, resulting in no change in LS and cardiac function, this is the reason why PIMSRA reduces myocardial contractility but does not significantly affect cardiac function. The purpose of Mavacamten, a cardiac myosin inhibitor, is to decrease heart contractility, which has been found to significantly improve LVOT obstruction.^28^ This highlights the importance of reducing contractility in the treatment of HOCM, and it is crucial to rely on this approach for the early reduction of LVOT in PIMSRA.

We noticed a considerable decline in SAM following the PIMSRA procedure. Unlike SM, PIMSRA does not have the ability to carry out MV repair during surgery,^29^, ^30^, ^31^ even though it is just as effective as SM in reducing the LVOT gradient.^32^, ^33^, ^34^ PIMSRA reduces SAM based on similar principles to Mavacamten. In theory, Mavacamten decreases myocardial contractility uniformly, leading to lowered RS, CS, and LS postmedication. Nonetheless, according to PIMSRA, diminishing strain in RS and CS solely can effectively alleviate or improve SAM‐induced MR. This finding holds more importance in safeguarding patient cardiac function and mitigating heart failure arising from reduced myocardial contractility.

Anatomic changes in the MV apparatus are a part of the pathological process of HCM. In recent years, their significant role in the obstruction process has been identified. The main and frequently observed characteristics include the elongation of MV leaflets, displacement of papillary muscles towards the anterior and base, especially the small anterior head of the anterolateral papillary muscle, and the shortening and thickening of chordae or insertion of the papillary muscle head into the mid‐anterior MV leaflet. All of these abnormalities contribute to the laxity of the mitral leaflets (resulting in decreased restriction to anterior movement) and protrusion of the leaflets into the LVOT, making them susceptible to being swept into the septum.^35^ The comparison between SAM patients and normal subjects who have HCM reveals that SAM patients have a smaller distance between the MV leaflet coaptation‐septal (C‐sept).^36^ As RS and CS weaken, there should be an increase in C‐sept, this may cause weakening or disappearance of the SAM phenomenon. It is uncertain if the presence of SAM affects patient lifespan following the decrease in LVOT gradient. The survival rates showed no significant difference between patients who underwent septal myectomy alone and those who had septal myectomy combined with MV surgery.^30^ Therefore, although there is no MV intervention during PIMSRA, it does not mean that it will affect the long‐term prognosis of patients.

The patient follow‐up revealed a significant improvement in symptoms of angina pectoris. However, there was no significant reduction in syncope, presyncope, or palpitation symptoms for the patients. Additionally, patients with heart function grade III–IV did not demonstrate any noteworthy improvement in heart function during the 30‐day follow‐up. We hypothesized that the improvement of syncope, palpitation, and heart function relied on the structural changes, which were confirmed in the long‐term follow‐up results of PIMSRA.^13^

For ERSA, the most crucial part of the procedure is accurately locating the contact point between the SAM and the IVS.^10^ Some studies noted a reduction of under 2 mm in the SAM area of the IVS,^37^ however, this small reduction was enough to disrupt the SAM‐septal feedback mechanism and effectively lower the LVOT, similar to ASA.^38^ Unlike SM, early improvement in obstructive conditions in ERSA does not occur due to structural changes in hypertrophic myocardium. Early studies on the behavior of LVOT obstruction during temporary balloon occlusion of the septal branch without alcohol injection support the hypothesis that the decrease of gradient may depend more on inducing a SAM localized reduction in contractility, the transient ischemia in septal branch at the site of obstruction already leads to an acute, reversible LVOT gradient reduction.^39^ After the ERSA, scar formation reduces the contractility in the SAM‐IVS. The endocardial myocardium typically exhibits the LS, therefore, the LS in the SAM region was weakened after ablation. The energy of radiofrequency ablation decreases with the blood flow, so it is reasonable to question the ablation depth. However, the scar on the endocardial myocardium weakens the LS sufficiently to reduce the gradient of LVOT. This recognition emphasizes the importance of changing the LS of IVS in patients with HOCM to achieve hemodynamic improvement. Future interventional ablation should focus on this, but with caution, to protect the cardiac function of patients.

After 30 days of follow‐up after the ERSA procedure, we observed a decrease only in LVOT pressure, without any improvement in MR, reduction in IVS thickness, or decrease in LAD. Moreover, there was no change in LVEF or in SAM. Similar to PIMSRA, we noticed a significant improvement in angina symptoms. The ERSA procedure exhibited less pericardial effusion in comparison to the PIMSRA group. This highlights the enhanced safety associated with the utilization of three‐dimensional mapping in the Carto system. The ERSA may be comparable to I and II area ablation and limited to the base or middle of the heart, it is important to consider if the reduction in contractility in this area is sufficient. This raises the question of the significance of entire IVS ablation. During the early ablation period of PIMSRA, the LVOT gradient decreased without thickness reduction, which was significantly greater than in the ERSA group. This highlights the vital role that the decreased contractile function of IVS plays. There was no difference in the gradient difference of the LVOT between the two groups before the procedure, as shown in Figure 2A. This finding suggests that there is no correlation between MR and gradient difference. There was no difference in preoperative LVEF between the PIMSRA group and the ERSA group. However, after the operation, the LVEF of the PIMSRA group was lower than that of the ERSA group. This difference was statistically significant, as depicted in Figure 2C. This indicates that ERSA did not alter the diastolic and systolic function of HOCM patients. Whether this change in only the gradient of LVOT can bring long‐term benefits still requires long time follow‐up. The PIMSRA group and the ERSA group showed no difference in preoperative LAD. However, postoperatively, the LAD in the PIMSRA group was lower than in the ERSA group, and this difference was statistically significant, as depicted in Figure 2D. This indicates that ERSA did not improve atrial remodeling, ERSA is unable to sufficiently reduce the LVOT gradient, thus limiting the improvement of MR and its impact on left atrial remodeling.^40^ For ERSA, further reducing LS of IVS may be a method to improve SAM, but the accompanying decrease in cardiac function may limit it, finding a balance point may be the future development direction of this procedure. Based on our analysis of Figure 2E,F, we have discovered a significant correlation between the reduction of MR and the decrease in LVOT, which is consistent with previous studies,^40^ at the same time accompanying the decrease of MR, left atrial remodeling is improved. As a result, it seems that the inability to reduce LVOT adequately is the reason why ERSA is not effective in reducing SAM‐mediated MR. Consequently, it would be worthwhile for future studies to investigate the specific pressure gradient that must be lowered to weaken or eliminate SAM. This could provide better guidance for HOCM ablation therapy.

## 5 LIMITATIONS

Our study is a retrospective examination concentrating on the new PIMSRA procedure, which has limited published research. The inclusion of a relatively small number of patients in our study suggests the necessity for larger clinical trials to verify the credibility of our findings. At our center, stress echocardiography (SE) is generally performed late, resulting in a lack of SE data for most patients. In the future, we aim to enhance the SE parameters for all patients diagnosed with HOCM.

## 6 CONCLUSIONS

In terms of the two types of radiofrequency ablation methods used in HOCM, it has been observed that PIMSRA demonstrates a more favorable early treatment effect compared to ERSA.

## AUTHOR CONTRIBUTIONS

All authors take responsibility for all aspects of the reliability and freedom from bias of the data presented and their discussed interpretation.

## CONFLICT OF INTEREST STATEMENT

The authors declare no conflicts of interest.

## Data Availability

The data on which the study is based were accessed from the Department of Cardiology, Zhengzhou No. 7 People's Hospital, the authors will supply the relevant data in response to reasonable requests.
